# From awareness to action: Health Belief Model-based educational intervention to improve breast self-examination practice among college teachers in Pakistan (CRCT)

**DOI:** 10.1371/journal.pone.0341804

**Published:** 2026-01-29

**Authors:** Benazir Mahar, Malina Binti Osman, Fatimah Ahmad Fauzi

**Affiliations:** Department of Community Health, Faculty of Medicine and Health Sciences, University Putra Malaysia, Selangor, Malaysia; Stony Brook University, UNITED STATES OF AMERICA

## Abstract

**Background:**

Breast cancer (BC) is the most common malignancy among women globally, with Pakistan showing similar trends. Teachers, especially female college teachers, are influential in health promotion. This study assessed the effectiveness of a Health Belief Model (HBM)-based educational intervention in improving breast self-examination (BSE) knowledge, beliefs, and practices.

**Methodology:**

A parallel cluster-randomized controlled trial was conducted among 114 female teachers from four colleges in Hyderabad, Pakistan, with colleges as the unit of randomization. The intervention group received multimedia presentations, survivor testimonials, pamphlets, breast models, gamified quizzes, and reminders, while the control group was offered the session after study completion. Data was collected at baseline, 1-month, and 3-month follow-ups using validated Urdu questionnaires. Analyses included descriptive statistics, bivariate tests, and Generalized Estimating Equations (GEE), using the Statistical Package for the Social Sciences (SPSS) version 29. A p-value < 0.05 was considered statistically significant.

**Results:**

Baseline characteristics were comparable across groups. Following the intervention, BSE practice improved significantly in the intervention group. At 1 month, participants were 5.7 times more likely to practice BSE (Adjusted Odds Ratio (AOR) = 5.724, p < 0.001), rising to 26.5 times at 3 months (AOR = 26.500, p < 0.001). Knowledge scores also increased significantly (p < 0.001). Significant between-group differences were observed in perceived susceptibility, severity, benefits, barriers, self-efficacy, and cues to action (all p ≤ 0.001).

**Conclusion:**

HBM-based intervention effectively enhanced BSE knowledge, beliefs, and practices while reducing barriers. Findings emphasize the value of structured educational interventions in promoting preventive health behaviors among female educators.

## Introduction

Breast cancer poses an escalating global health burden, particularly in low-resource settings such as Pakistan, where it ranks as the most prevalent malignancy among women. According to the annual cancer report by the International Cancer Registry, breast cancer accounted for 51% of all female cancer cases recorded in 2022 in Pakistan [1]. Similarly, in Pakistan, a study reported that 89% of patients receive a delayed diagnosis, and 59% of those individuals have an advanced diagnosis [2]. Another study conducted in a tertiary care center to determine the stage of diagnosis revealed that only 4.6% of patients were found in stage 1, 47.26% came from stage 2; however, the rest were diagnosed at later stages [3]. Effective treatment of breast cancer also depends on early diagnosis, which can lower the death rate. High mortality associated with breast cancer is often attributed to late-stage diagnoses [4,5].

In the case of breast cancer, delayed diagnosis is largely attributed to a profound lack of awareness and the underutilization of screening practices [6]. Numerous previous studies have identified a lack of awareness as a major barrier to early diagnosis [7]. The adoption of preventive practices is further hindered by social barriers, misinformation, and a lack of health education, despite the proven effectiveness of early detection in reducing mortality [8]. Breast cancer awareness, clinical breast examination (CBE), and diagnostic imaging, such as ultrasound and mammography, play an important role in the pathway to breast cancer detection [9]. In Pakistan, breast health assessment relies largely on opportunistic or diagnostic methods rather than organized screening. Clinical breast examination (CBE) is commonly performed in healthcare settings, while ultrasound is mainly used for diagnostic evaluation, particularly among younger women [10]. In contrast, mammography is the only established population-level screening modality; however, its availability and uptake are extremely limited due to the lack of a national screening program, high costs, and restricted access to functional mammography units [11]. These constraints contribute to delayed diagnosis and highlight the importance of strengthening breast awareness behaviors in low-resource settings. The effectiveness of BSE is a topic of debate. Breast self-examination was not a reliable method of screening for breast cancer, according to the major randomized studies conducted in Shanghai [12]. Similarly, the Canadian Task Force on Preventive Health Care and the US Preventive Services Task Force concluded that women no longer benefit from breast self-examinations [13]. Although BSE alone is not sufficient for early detection of breast cancer, it is an effective tool for raising breast cancer awareness [14] and providing the opportunity to educate women about breast cancer in developing and underdeveloped countries like Pakistan. Moreover, the BSE training and adherence are a gateway to health promotion behavior that gives women knowledge and sets for adherence to clinical breast examination (CBE) and mammography screening guidelines later in life [15]. In resource-limited settings like Pakistan, the practical relevance of BSE increases because it promotes basic breast awareness in contexts where access to organized screening services remains limited [16,17].

It is important to comprehend what women may or may not know about breast cancer to raise breast cancer awareness among them and foster their trust in the BSE procedure. We also need to comprehend their attitudes toward early diagnosis of breast cancer, the advantages and disadvantages of BSE practice, and other screening techniques [18].

The Health Belief Model (HBM) is one of the most popular conceptual frameworks that is frequently utilized as an instructional program [19]. The Health Belief Model (HBM), an extensively validated theoretical framework, offers valuable insights into health-related decision-making by focusing on individuals’ perceptions of risk, benefits, and self-efficacy [20]. Leveraging this model, educational interventions can effectively recalibrate beliefs and attitudes, thus fostering sustained behavioral change in preventive health practices. Furthermore, Health belief plays an important role in an individual’s interest in health protection behavior, which leads to screening practices in different countries and cultures [21].

This model emphasizes that health behavior is affected by threats from health problems; for example, women who perceive susceptibility to breast cancer risk or believe that breast cancer is a serious disease are more likely to do the BSE. Women with higher health motivation, who perceive greater benefits and feel fewer barriers to breast examination, are more likely to perform BSE [22]. In this study, each component of the educational intervention was intentionally mapped to the HBM constructs. Information on breast cancer risk and consequences addressed perceived susceptibility and severity, while the structured BSE training aimed to enhance self-efficacy. Interactive discussions and demonstrations helped reduce perceived barriers, and reminder tools (brochures, digital messages, branded items) functioned as cues to action. This alignment ensured that the intervention directly operationalized the HBM pathways expected to influence BSE behavior.

Several interventions have been implemented globally to improve women’s BSE practices and breast cancer screening rates [23]. A group of university students in Bangladesh, aged 18–26, showed significant improvements in breast cancer knowledge and BSE practices after an educational intervention. Post-test mean scores increased significantly for breast cancer prevention (3.82 to 7.14), screening (1.82 to 3.98), and BSE process (1.57 to 3.94) (all p < 0.001). Additionally, BSE practice improved significantly from 21.3% to 33.8% (p < 0.001) [24]. Nevertheless, no randomized controlled study has been conducted in Pakistan to raise teachers’ knowledge of BSE practices and breast cancer. Teachers represent an important yet under-addressed population in breast health promotion because of their unique ability to influence health literacy within families, educational institutions, and the broader community. As trusted and educated professionals, they often serve as information gatekeepers and can effectively disseminate accurate health messages to students and their social networks. Improving BSE practices and strengthening breast health awareness among teachers may therefore yield a wider community impact by encouraging early detection behaviors and fostering a proactive, health-seeking culture. Despite this potential, there remains a dearth of research on interventions specifically designed to improve teachers’ screening practices and awareness of breast cancer in Pakistan. To address this gap, this cluster-randomized controlled trial (CRCT) evaluates the effectiveness of a comprehensive HBM-based educational intervention aimed at improving breast health awareness and BSE adherence among college teachers in Hyderabad, Pakistan.

By employing a multifaceted intervention strategy, including interactive presentations, experiential learning, and continuous reinforcement, addressing common myths and misbeliefs, this study endeavors to bridge critical knowledge gaps, dismantle misconceptions, and empower educators to take charge of their breast health, thereby contributing to early detection and potentially reducing breast cancer-related morbidity and mortality in the region.

### Study objectives

To develop, validate, and implement a health educational intervention grounded in the Health Belief Model (HBM), and to evaluate its effectiveness in enhancing breast self-examination (BSE) practices, as well as knowledge and beliefs about breast cancer and BSE among female college teachers in Hyderabad.

**Fig 1:**
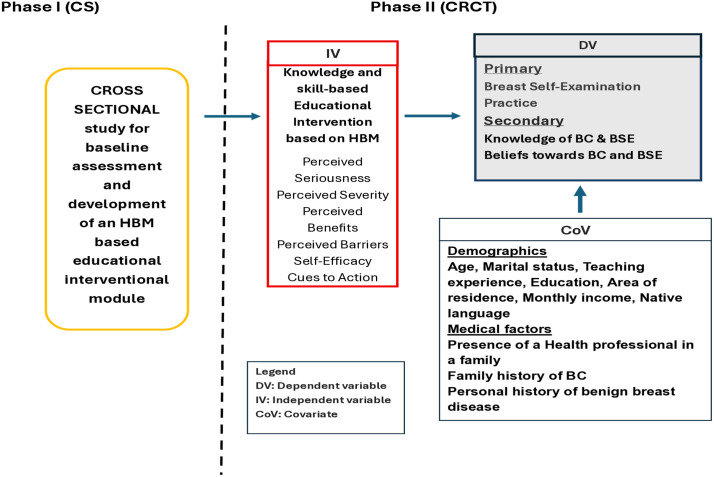
Conceptual framework of the study.

To compare BSE practices (Primary outcome), knowledge, and beliefs (secondary outcome) alongside socio-demographic and medical characteristics between intervention and control groups at baseline, one month, and three months post-intervention, and to analyze within- and between-group differences over time after adjusting for relevant covariates (Fig 1).

## Materials and methods

### Administrative information

The protocol for this CRCT is registered with the **Thai Clinical Trial Registry (TCTR),** TCTR20240703005 (https://www.thaiclinicaltrials.org/show/TCTR20240703005) [25].

### Study area, design, and setting

The recruitment period for this study was from September 1, 2024, to December 15, 2024. The study was conducted in Hyderabad, Pakistan, the eighth-largest city located in the Sindh province. It serves as a gateway to interior Sindh and is situated approximately 150 kilometers northeast of the provincial capital, Karachi. This research employed a single-blind, parallel cluster-randomized controlled trial (CRCT), with government girls’ colleges in Hyderabad, Pakistan, serving as the unit of randomization and teachers as study participants. The primary aim was to assess the impact of a health belief model-based educational intervention on breast self-examination (BSE) knowledge, beliefs, and practices among female college teachers. The trial adhered to the Consolidated Standards of Reporting Trials (CONSORT) guidelines for cluster-randomized trials. Among the ten eligible government girls’ colleges in the Hyderabad district, four were selected for the intervention phase of this CRCT. The intervention group received a structured educational program on breast cancer and BSE, while the control group was given the same educational content upon the study’s completion. Evaluations were carried out at baseline, and at one- and three-month post-intervention.

### Sample size calculation

The sample size was calculated based on the expected difference in BSE practice between the intervention and control groups. By using the Two Proportion Formula,


$$\frac{𝐧\;=\;[𝐳(1-\alpha /2)\;\sqrt{}2𝐏\overset{―}{}(1-𝐏\overset{―}{})\;+\;𝐳(1-\beta )\;\sqrt{}({𝐏}_{1}(1-{𝐏}_{1})\;+\;{𝐏}_{2}(1-{𝐏}_{2}))²}{({𝐏}_{1}\;-\;{𝐏}_{2})\;²\;}$$

Drawing on prior literature, the assumed baseline proportion of BSE practice was 86.5% for the intervention group and 26% for the control group [26]. Using a power of 80% and a significance level of 0.05, the estimated sample size was 42 participants per group. The design effect (DE = 1.9) was calculated using the formula DE = 1 + (m – 1) × ICC, where *m* was the average cluster size 99, and the ICC was estimated at 0.02 based on similar educational intervention studies. Using these values, the resulting DE was approximately 1.9. After adjusting to a 20% attrition rate and 90% eligibility rate, the sample size was increased to 57 participants per group, totaling 114 participants.

### Sampling method

**Fig 2:**
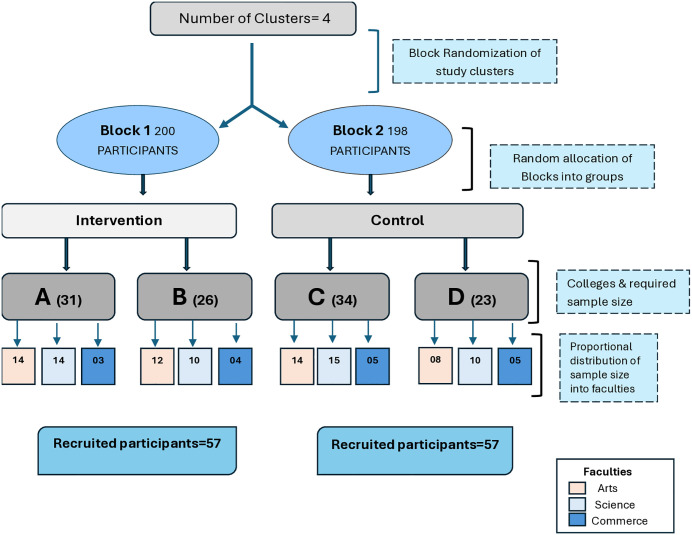
Outline of the study sampling.

We conducted a two-arm, parallel, cluster-randomized controlled trial with a 1:1 allocation ratio at both the cluster (2 colleges per arm) and participant levels (57 participants per arm; total N = 114). Randomization was performed at the college (cluster) level using blocked allocation to ensure balance across arms. A multi-stage sampling approach was implemented. Initially, the eligibility of colleges was assessed based on infrastructure, teacher availability, and willingness to participate. Of the 10 eligible institutions, 6 were utilized in the pretesting and cross-sectional phases, while the remaining 4 were reserved for the CRCT phase. These 4 colleges were stratified into two blocks based on faculty size and then randomly allocated to intervention or control arms using simple randomization (coin toss method). Proportionate stratified sampling was then employed within each college based on faculty composition and individual teachers were randomly selected using a random number generator, as shown in Fig 2

### Eligibility criteria

Colleges included were the government colleges in Hyderabad, and they agreed to participate. Colleges were excluded if they had participated in earlier phases of the study (pilot, pretesting, or cross-sectional survey). Of the 10 eligible colleges, six were used exclusively for pretesting/cross-sectional phases to avoid contamination, leaving four comparable institutions for randomization. Although only four colleges were available for randomization, the average cluster size was large, and the design effect (DE = 1.9) ensured adequate power for detecting intervention effects.

Eligible participants were female teachers aged 25–59 years, currently employed at selected colleges, and willing to provide informed consent. Teachers were excluded if they were breast cancer patients or survivors, pregnant or lactating, or on extended leave during data collection.

### Recruitment of participants

**Fig 3:**
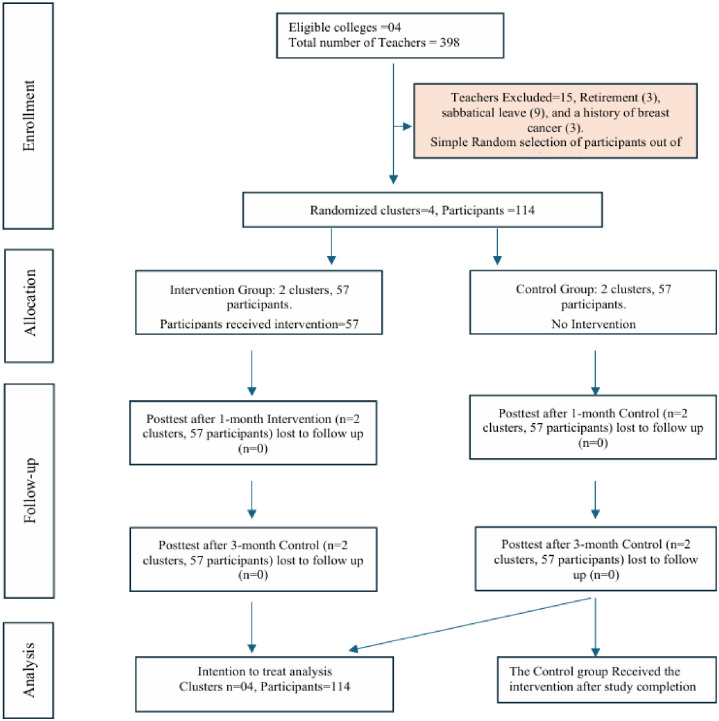
CONSORT flow diagram of the study.

After obtaining formal permission from the college administration, meetings were arranged with the help of designated coordinators. The researcher briefed eligible teachers about the study objectives, benefits, and participation criteria. Written informed consent was obtained from those willing to participate. Selected teachers then completed the baseline questionnaire, after which the intervention was delivered to the intervention group. Follow-up assessments were conducted at one- and three-month post-intervention, using the same questionnaire. All responses were anonymized, and participants were encouraged to answer honestly (Fig 3). The same group was followed across baseline, 1-month, and 3-month assessments, resulting in a longitudinal (panel) dataset within each cluster.

### Randomization procedure

Cluster randomization was performed at the college level. The four selected colleges were grouped into two blocks to ensure balance in faculty size. Each block was randomly allocated to either the intervention or control group using the coin toss method. To maintain blinding, teachers were informed that they were participating in a study on breast self-examination but were unaware of their group assignment. The same researcher delivered the intervention in both assigned colleges to maintain consistency.

### Development of educational intervention

An interventional module was developed based on the SIDEK Model of Instructional Design, incorporating evidence-based guidelines from the American Cancer Society (ACS) and conceptually grounded in the Health Belief Model (HBM) to enhance breast cancer (BC) awareness and promote breast self-examination (BSE) practices.

a **Goals of the Health Education Module**

The primary goals of the health education module were to improve participants’ knowledge of breast cancer symptoms, risk factors, and the importance of early detection. It also aimed to increase the frequency and accuracy of breast self-examination (BSE) practices, address common barriers and misconceptions related to breast cancer and BSE, and foster a proactive and informed approach toward breast health and early detection behaviors.

b **Module Development**

The health education package was strategically developed to address knowledge deficits and encourage positive behavioral change regarding breast cancer awareness and breast self-examination (BSE). It comprised a series of evidence-based, culturally relevant components, including educational videos of a breast cancer survivor’s testimony, and an instructional BSE video in Urdu to provide both emotional engagement and practical guidance. A visually engaging leaflet, featuring illustrations and concise information on breast cancer symptoms, risk and protective factors, and the correct technique for BSE, was distributed for continued reference. To reinforce learning, participants engaged in an interactive, gamified quiz assessing their understanding of BSE. The educational session also featured a PowerPoint presentation highlighting key facts about breast cancer and step-by-step BSE instructions. A hands-on BSE demonstration using breast models allowed participants to practice the technique under supervision, with an accompanying Q&A session to address concerns and clarify doubts. Additionally, digital reminders were sent post-intervention to sustain motivation and promote adherence to regular BSE practices (Table 1).

**Table 1:** Outline of an intervention plan.

Session	Learning objectives	Target Area	HBM constructs
Breast Structure, Function, and Cancer Overview	Recognize breast cancer types and high-risk groups. Identify key signs and symptoms,	Knowledge on BC	Perceived Susceptibility Perceived severity
Knowledge of BC screening methods	Understand the importance of early breast cancer detection. Familiarize yourself with various breast cancer screening methods.	Knowledge & Beliefs on BC Screening Methods	Perceived benefits Perceived barriers
Breast Health Awareness and BSE	Understand the basics of breast health and FPBAC. Learn the timing and importance of performing BSE.	Knowledge and beliefs on breast health awareness	Perceived benefits Perceived barriers. Motivation Self-efficacy
Guidance on BSE performance	Master the steps and correct pressure levels for effective BSE. Increase confidence and motivation to perform regular BSE.	Knowledge and beliefs on breast health awareness. Practice of BSE Practice of BSE	Perceived benefits Perceived barriers Cues to action
BSE practice on the model	Demonstrate proper BSE technique	Practice of BSE	Perceived benefits & barriers, cues to action, Self-efficacy
Frequently Asked Questions on BC and BCS	To clarify common misconceptions and provide accurate information regarding BC and its screening. To reinforce knowledge about the importance of early detection and regular screenings.	Knowledge and beliefs related to BC and screening	Perceived Susceptibility Perceived Benefits Perceived Barriers

### Intervention delivery

The module was delivered face-to-face in two intervention colleges. In the first college, 34 participants attended the session, while the second college hosted 23 participants. Each session lasted approximately 2 hours and 50 minutes (excluding breaks), incorporating multimedia presentations, skill-based demonstrations, and interactive discussions.

The intervention consisted of an educational PowerPoint presentation highlighting key facts about breast cancer, the importance of early detection, and the role of regular breast self-examination (BSE). This was followed by a skill-based session where participants received practical demonstrations and were invited to practice BSE on human breast models to enhance their understanding. To reinforce learning, a gamified quiz based on the BSE procedure was conducted, promoting active engagement and knowledge retention. Participants were also provided with educational brochures for reference and were informed about the importance of regular BSE practice. The session concluded with an interactive segment, including a discussion and Q&A to address participants’ queries. For the control group, the same educational content and practical training were provided after the final follow-up data collection.

a **Ensuring Intervention Fidelity**

To maintain standardization and fidelity across sessions, the intervention was delivered using a uniform protocol developed by the principal investigator. Role-play and demonstration techniques were employed during BSE practice sessions to enhance participant engagement and comprehension. Additionally, brief implementation notes were recorded during each session to confirm that the planned content and sequence were being consistently followed. Additional reinforcement tools, including branded materials and digital reminders, were used to reiterate key health messages and encourage sustained BSE practice.

### Questionnaires used for data collection

#### Section I: Sociodemographic and Medical History.

This section gathers data on participants’ age, teaching experience, income, marital status, religion, education level, residence, native language, and the presence of a medical professional in the family. It also records a brief medical history, including personal history of benign breast conditions and family history of breast cancer.

#### Section II: Knowledge of Breast Cancer and Breast Self-Examination (BSE).

Comprising 36 items, this section assesses participants’ knowledge across four domains: risk factors (13 items), symptoms (8 items), screening modalities (4 items), and BSE (11 items). Responses are scored as 1 for correct answers, with a total score range of 0–36. Higher scores indicate greater knowledge.

#### Section III: Beliefs About Breast Cancer and BSE (Urdu CHBMS).

This section employs the Urdu version of Champion’s Health Belief Model Scale (CHBMS) to assess beliefs related to breast cancer and BSE. It includes 32 items rated on a 5-point Likert scale and covers six constructs: perceived susceptibility (3 items), severity (6), benefits of BSE (4), barriers (4), self-efficacy (8), and health motivation (7). Higher scores reflect stronger health beliefs, except for perceived barriers, which are inversely related to screening behavior.

#### Section IV: BSE Practice.

BSE practice was assessed using Section IV of the questionnaire, which contained two items specifically designed to capture both engagement in the behavior and its frequency. The first item measured self-reported BSE practice using a binary response format (Yes/No), indicating whether the participant had performed BSE within the specified recall period. The second item evaluated the frequency of practice on an ordinal 4-point scale (1 = Never, 2 = Once a month, 3 = Once in three months, 4 = Once in six months).

a **Quality Control of Instruments**

The original CHBMS-BSE consisted of 42 items across six subscales: perceived severity (5 items), susceptibility (7), benefits (6), barriers (6), confidence (11), and motivation (7). Content validity was assessed by four experts from the Community Health Department, UPM, using a 4-point relevance scale. Items with an I-CVI ≥ 0.80 were retained, while those below the threshold were revised or removed based on expert feedback.

For cultural adaptation to the Pakistani context, a standardized forward-backward translation procedure was employed. Two bilingual experts translated the scale into Urdu, followed by back-translation by independent linguists. A multidisciplinary panel reviewed both versions to ensure semantic and contextual accuracy. Based on expert consensus, four redundant items were removed, resulting in a refined 32-item Urdu version (U-CHBMS).

Reliability analysis showed substantial to almost perfect inter-rater agreement (κ = 0.61–0.80 and >0.80), particularly for BSE-related items. Internal consistency was high across all subscales, with Cronbach’s alpha values ranging from 0.80 to 0.90, indicating excellent reliability.

### Data analysis

Data was analyzed using IBM SPSS Statistics version 29.0, with statistical significance set at p < 0.05. Chi-square tests were used to assess baseline group differences for categorical variables, while the Mann–Whitney U test and independent t-test were applied for continuous variables. Generalized Estimating Equations (GEE) were employed as a robust analytic method suitable for clustered and longitudinal data. This approach allowed for the assessment of between-group effects (e.g., college), within-group temporal changes, and group-by-time interaction effects. GEE also adjusts for intra-cluster correlation and does not depend on strict distributional assumptions. Robust (sandwich) standard errors were applied to ensure valid inference even when normality assumptions were not met. BSE practice (binary) was modeled using a binomial family with a logit link function, whereas continuous outcomes (knowledge and beliefs) were analyzed using a Gaussian family with an identity link. An exchangeable working correlation structure was used across all models to account for within-cluster correlation among teachers from the same college.

### Ethical clearance

Ethical clearance for the study was granted by the Ethical Review Board of Mekran Medical College, Pakistan (Ref No MMC/ERC/1/6/24). Additional approval and administrative permission were obtained from the College Education Department, Sindh (Ref No DCEHRH-2023–24/423). Before data collection, written informed consent was obtained from all participants. The study’s objectives and potential benefits were communicated, and participants were informed of their right to decline participation or withdraw at any stage without any repercussions. To uphold confidentiality, anonymized code numbers were used to identify colleges and participants in place of personal identifiers.

## Results

### Response rate and normality assessment

A total of 114 teachers participated in the study, with 57 randomly allocated to the intervention group and 57 to the control group. Normality of the data was assessed using both the Kolmogorov-Smirnov and Shapiro-Wilk tests. The results indicated that the majority of the variables, knowledge, perceived susceptibility, perceived severity, perceived benefits, perceived barriers, and cues to action, were not normally distributed, as evidenced by significant p-values (p < 0.05) in both tests. Only the self-efficacy variable demonstrated normality (Shapiro-Wilk p = 0.135).

### Comparison of socio-demographic variables between the intervention and control groups at Baseline

A comparison of socio-demographic characteristics between the intervention and control groups revealed no statistically significant differences across variables such as age, teaching experience, monthly family income, marital status, religion, education level, area of residence, and native language (Table 2), Additionally, both groups were comparable in terms of having health professionals in the family, a family history of breast cancer, and a personal history of benign breast disease. These results indicate that the two groups were demographically balanced at baseline.

**Table 2:** Socio-demographic variables between the intervention and control groups at Baseline.

Characteristics	Intervention (n = 57) n (%)	Control (n = 57) n (%)	Test Statistic	P- value
**Age** (Median IQR)	40(12)	40(14)	U = 1387.5	0.17
**Teaching experience** Median (IQR)	13(12)	13(10)	U = 1508.5	0.51
**Family Income**				
Middle	33(57.9)	38(66.7%)	0.93	0.33
High	24(42.1)	19(33.3%)		
**Marital status**				
Single	9(15.8)	10(17.5)	0.74	0.6
Married	44(77.2)	45(78.9)		
Divorced/separated/widowed	04(7.0)	02(3.5)		
**Religion**			---	1.0
Muslim	54(94.7)	55 (96.5)		
Non-Muslim	3(5.3)	02(3.5)		
**Education level**				
Graduate	02(3.5)	00	1.71	0.65
Masters	50(87.7)	52(91.2)		
Doctorate	05(8.8)	05(8.8)		
**Area of residence**				
Rural	01(1.8)	01(1.8)	0.51	1.0
Urban	53(93.0)	54(94.7)		
Suburb	03(5.3)	02(3.5)		
**Native language**				
Sindhi	39(68.4)	40(70.2)	1.47	0.9
Urdu	15(26.3)	13(22.8)		
Others	03(5.3)	04(7.1)		
**The presence of health professionals in the family**				
Yes	36(63.2)	37(64.9)	0.03	0.8
No	21(36.8)	20(35.1)		
**Family history of breast cancer**				
Yes	13(22.8)	14(24.6)	0.04	0.8
No	44(77.2)	43(75.4)		
**Personal history of benign breast diseases**				
Yes	05(8.8)	04(7.0)	--	1.0
No	52(91.2)	53(93)		

**U=Mann-Whitney test, t=T test**

**Table 3** presents the baseline comparison of breast self-examination (BSE) practice, knowledge, and Health Belief Model (HBM) domains between the intervention and control groups. At baseline, no statistically significant differences were observed between the groups in terms of BSE practice rates or frequency, indicating a comparable starting point for both groups. Similarly, median scores for knowledge, perceived susceptibility, perceived severity, perceived benefits, perceived barriers, and cues to action, as well as mean self-efficacy scores, were also statistically non-significant between the two groups (all p > 0.05). These findings confirm that both groups were demographically and behaviorally equivalent at the outset of the study, ensuring the internal validity of subsequent intervention effect assessments.

**Table 3:** Baseline Comparison of BSE Practice, Knowledge, and Beliefs Between Groups.

Variable	Intervention (n = 57)	Control (n = 57)	Test Statistic	P- value
**Performing BSE (Yes)**	19 (33.3%)	16 (28.1%)	χ² = 0.37	0.5
**Performing BSE (No)**	38 (66.7%)	41 (71.9%)		
**BSE Frequency (Regular)**	3 (15.7%)	5 (31.2%)	χ² = 1.61	0.6
**BSE Frequency (Irregular)**	16 (84.2%)	11 (68.7%)		
**Knowledge**	17.0 (3.0)	17.0 (5.0)	U = 1591.5	0.8
**Perceived Susceptibility**	9.0 (1.5)	9.0 (2.0)	U = 1559.0	0.7
**Perceived Severity**	18.0 (3.0)	18.0 (3.5)	U = 1580.0	0.7
**Perceived Benefits**	14.0 (2.0)	13.0 (1.0)	U = 1346.5	0.1
**Perceived Barriers**	12.0 (2.0)	12.0 (2.0)	U = 1618.5	0.9
**Self-Efficacy**	28.9 ± 3.47	28.9 ± 3.07	t = 0.00	1.0
**Cues to Action**	24.4 ± 2.54	24.5 ± 2.54	U = 1559.0	0.7

U=Mann-Whitney test, t=T test

### Generalized estimating equations (GEE) analysis of changes in the primary response variable BSE practice between study groups across timepoints, and the interaction between time and group

Table 4 assessing the impact of the intervention on breast self-examination (BSE) practice over time, accounting for both group differences and group-by-time interactions. The overall group effect indicated that participants in the intervention group were 1.28 times more likely to perform BSE compared to those in the control group; however, this difference was not statistically significant (AOR = 1.282, p = 0.54). Regarding the effect of time alone, no significant changes in BSE practice were observed at either the 1-month follow-up (AOR = 0.757, p = 0.20) or the 3-month follow-up (AOR = 1.0, p = 1.0). In contrast, the interaction between group and time demonstrated a significant effect. At the 1-month follow-up, participants in the intervention group were 5.7 times more likely to practice BSE than those in the control group (AOR = 5.724, p < 0.001), and this likelihood increased substantially at the 3-month follow-up, with intervention participants being 26.5 times more likely to engage in BSE (AOR = 26.500, p < 0.001).

**Table 4:** GEE of BSE practice (Primary outcome) between groups, across time points, and the interaction between time and group.

Variables	β	SE	AOR	95% CI (lower-upper)	p-value
Intercept	−0.941	0.294			
**Group**					
Control	Ref				
Intervention	0.248	0.407	1.282	0.577-0.284	0.54
**Timepoint**					
Baseline	Ref				
1-Month	−0.278	0.243	0.757	0.470-1.220	0.2
3-Month	−3.077	0.274	1.0	0.584-1.714	1.0
**Group × Timepoint**					
Control × Baseline	Ref				
Intervention × 1 Month	1.745	0.3941	5.724	2.644-12.391	<0.001*
Intervention × 3 months	3.277	0.605	26.500	8.087-86.83	<0.001*

SE: Standard error, CI: Confidence interval, AOR: Adjusted odds ratio.

**Table 5** presents the results of the Generalized Estimating Equations (GEE) analysis assessing breast self-examination (BSE) practice between and within the intervention and control groups over time. The between-group comparison shows that participants in the intervention group were significantly more likely to practice BSE compared to those in the control group. Within-group analysis further reveals a significant increase in BSE practice at both 1-month and 3-month follow-ups in the intervention group, while no significant change was observed in the control group. These findings indicate the effectiveness of the educational intervention in promoting regular BSE practice.

**Table 5:** GEE Analysis of BSE Practice (Between and Within Groups).

Group	Timepoint	β	SE	AOR	95% CI	P-value
**Between groups**						
Intervention vs. Control	-------	1.532	0.308	4.629	2.527–8.478	<0.001*
**Within Groups**						
Intervention	1-month follow-up	1.466	0.309	4.333	2.361–7.955	<0.001*
Intervention	3-month follow-up	3.277	0.539	26.500	9.202–76.31	<0.001*
Control	1-month follow-up	−0.278	0.243	0.757	0.470–1.220	0.200
Control	3-month follow-up	−5.870	0.274	1.000	0.584–1.714	1.000

SE: Standard error, CI: Confidence interval, AOR: Adjusted odds ratio.

**Table 6** presents the percentage of participants performing breast self-examination (BSE) in the intervention and control groups at baseline, 1-month, and 3-months. Within-group changes from baseline are shown for both follow-up points, along with the difference-in-change, representing the absolute effect attributable to the intervention compared with the control group.

**Table 6:** Absolute effect size of BSE practice across baseline, 1-month, and 3-month follow-up.

Group	Baseline (%)	1-Month (%)	Change Baseline (1-Month)	3-Month (%)	Change Baseline (3-Month)
**Intervention**	33.3	68.4	+35.1%	93.0	+59.7%
**Control**	28.1	22.8	–5.3%	28.1	0%
**Difference-in-Change**	–	–	+40.4%	–	+59.7%

### Generalized estimating equations (GEE) analysis of changes in secondary response variables, knowledge, and beliefs between groups, across time points, and the interaction between time and group

**Table 7** summarizes the findings from the Generalized Estimating Equations (GEE) analysis used to evaluate the effects of the intervention on knowledge and Health Belief Model (HBM) constructs over time. Although the independent effects of group and time were not statistically significant for most variables, the group-by-time interaction effects were significant across all constructs. This indicates that the educational intervention led to significant improvements in participants’ knowledge, perceived susceptibility, severity, benefits, self-efficacy, and cues to action related to breast cancer screening over the follow-up period.

**Table 7:** GEE of knowledge and HBM constructs between groups, across time points, and the interaction between time and group.

Variable	Group Effect	Time Effect	Group × Time Interaction
**Knowledge**	χ² (1) =0.15, p = 0.69	χ² (2) =2.55, p = 0.27	χ² (2) =4778.92, p < 0.001*
**Perceived Susceptibility**	χ² (1) =0.35, p = 0.55	χ² (2) =6.51, p = 0.06	χ² (2) =312.51, p < 0.001*
**Perceived Severity**	χ² (1) =0.07, p = 0.786	χ² (2) =8.83, p = 0.013*	χ² (2) =521.85, p < 0.001*
**Perceived Benefits**	χ² (1) =2.71, p = 0.10	χ² (2) =2.70, p = 0.26	χ² (2) =214.40, p < 0.001*
**Perceived Barriers**	χ² (1) =0.004, p = 0.952	χ² (2) =2.66, p = 0.26	χ² (2) =218.90, p < 0.001*
**Self-Efficacy**	χ² (1) =0.00, p = 1.00	χ² (2) =6.06, p = 0.048*	χ² (2) =178.73, p < 0.001*
**Cues to Action**	χ² (1) =0.14, p = 0.710	χ² (2) =1.04, p = 0.594	χ² (2) =165.99, p < 0.001*

### GEE of Knowledge and Beliefs Domains Between the Intervention and Control Groups

**Table 8** displays findings from the Generalized Estimating Equations (GEE) analysis conducted to evaluate differences in knowledge and Health Belief Model (HBM) domains between the intervention and control groups. Each domain was assessed to determine the impact of the educational intervention on participants’ breast cancer-related knowledge and beliefs.

**Table 8:** GEE of Knowledge and Beliefs Domains Between the Intervention and Control Groups.

Domain	β	95% CI	p-value
**Knowledge**	8.142	7.21–9.07	<0.001*
**Perceived Susceptibility**	1.924	1.477–2.371	<0.001*
**Perceived Severity**	3.129	2.286–3.971	<0.001*
**Perceived Benefits**	1.602	1.158–2.047	<0.001*
**Perceived Barriers**	2.263	1.757–2.770	<0.001*
**Self-Efficacy**	2.511	1.493–3.529	<0.001*
**Cues to Action**	2.278	1.510–3.045	<0.001*

CI: Confidence interval

A statistically significant increase was observed in the intervention group compared to the control group across all assessed domains. Specifically, the intervention group showed markedly higher knowledge scores (β = 8.142, p < 0.001), indicating a substantial improvement in understanding of breast cancer and screening practices. Similarly, all belief-related constructs perceived susceptibility, perceived severity, perceived benefits, perceived barriers (reverse scored), self-efficacy, and cues to action were significantly enhanced in the intervention group (all p-values < 0.001).

**Fig 4:**
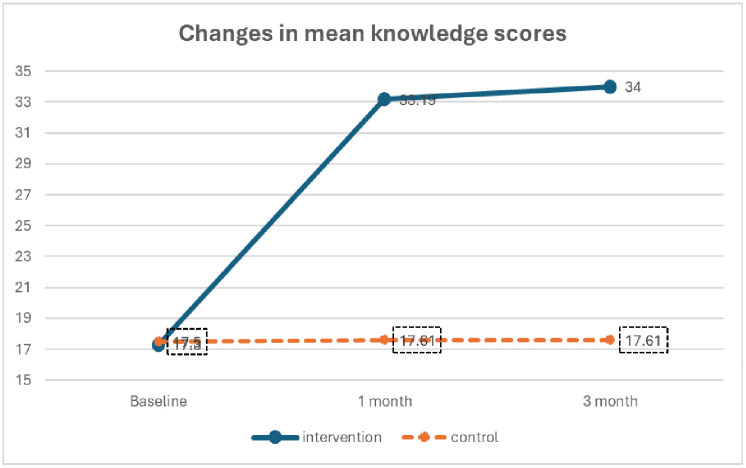
Change in Knowledge scores over time.

**Fig 4** illustrates the progression of mean knowledge scores across three time points: baseline, 1-month follow-up, and 3-month follow-up for both the intervention and control groups. In the intervention group, a marked increase was observed, with scores rising from 17.55 at baseline to 33.19 at 1 month, and further to 34.00 at 3 months, reflecting a sustained enhancement in knowledge. Conversely, the control group exhibited minimal change, with scores remaining virtually unchanged at 17.56 at baseline, 17.61 at 1 month, and 17.61 at 3 months.

**Fig 5:**
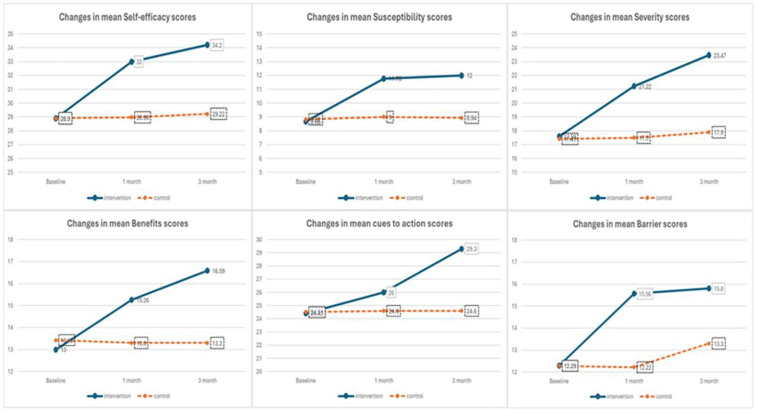
Changes in belief scores across time points.

**Fig 5** illustrates the changes in mean scores of Health Belief Model (HBM) constructs self-efficacy, susceptibility, severity, benefits, cues to action, and barriers at baseline, 1-month, and 3-month follow-ups for both intervention and control groups. Notably, the intervention group exhibited marked improvements across all constructs over time. Self-efficacy scores rose significantly from 28.53 at baseline to 34.2 in three months, while perceived susceptibility increased from 9.1 to 12. Similarly, severity scores improved from 18.47 to 23.47, and perceived benefits rose from 13.19 to 16.59. Cues to action it showed a notable increase from 24.63 to 29.3. Although perceived barriers also increased from 12.29 to 15.8, this may reflect heightened awareness rather than a negative outcome. In contrast, the control group’s scores remained relatively stable across all constructs. These trends highlight the positive and sustained impact of the educational intervention on modifying health beliefs related to breast cancer screening.

## Discussion

### Demographic, social, and health-related Attributes of Study Participants

Demographic, social, and health-related attributes of participants in both groups were broadly comparable at baseline. The age distribution in our sample aligns with findings from similar studies by Torbaghan et al. [27]. Among teachers and working women in LMICs, where participants typically fall within the mid-30s to early-40s age range, Consistent with previous research from the Philippines, [28]. and Saudi Arabia by Kandasamy et al [29]. Most teachers in our study were married and had substantial professional experience. Educational attainment was generally high, which is expected for college faculty. However, unlike studies from Pakistan and neighboring regions [30,31], where bachelor’s degrees were more common, our participants predominantly held master’s qualifications.

The socioeconomic profile of participants also aligned with the typical middle-income status of educators in urban Pakistan. Overall, these demographic patterns are consistent with published literature involving teacher populations, supporting the representativeness of our sample and strengthening the internal validity of the trial. Unlike Rakhshani et al [32], who reported that most participants earned less than 100 USD per month and lie in the lower income category when considering the World Bank ranking.

A significant majority of respondents identified as Muslim, but a minority reported as non-Muslim. This religious distribution aligns with Pakistan’s demographic structure, where Islam is the predominant religion. The majority of teachers lived in urban areas. This distribution aligns with the findings of Liu et al. (2010), who reported that approximately 74% of participants in their community-based BSE intervention in China were from urban or suburban areas, while 26% represented rural communities [33].

### Health and Family-Related Characteristics

In this survey, 63.2% of the treatment group and 64.9% of the control group reported having a health professional within their family. Consequently, in terms of family history, 22.8% of participants in the intervention and 24.6% in the control group reported a familial history of BC. This frequency exceeds the figures published by Shoushtari and Khayali et al, which reported family histories of 6% and 6.5%, respectively [34,35]. However, it is slightly lower than the 30.2% reported by Emami et al [36].

Only 7.9% of participants reported a previous exposure to benign breast conditions, whereas the majority (92.1%) revealed no such history. These findings support the results reported by Ashtarian et al., who found 3.57% with a previous history of breast masses [37]. However, a study reported by Seyed et al reported an increased prevalence of 10.5% of personal history of benign breast diseases [38].

### Baseline Group Differences

The present study demonstrated differences in respondents’ characteristics and outcome variables between the groups at baseline; however, these differences were not statistically significant. This aligns with the findings of Goel and O’Connor [39], Noman et al., and Martin et al. [30,40], which demonstrated no statistically significant variations in variables among groups at baseline. The findings suggest that, while minor changes may exist, they do not reach a level of significance that warrants further investigation at the baseline stage.

### Breast Self-Examination (BSE) Practices

In this study, only 30.7% of participants indicated that they practice breast self-examination (BSE), whereas the majority, 69.3%, reported that they do not engage in this activity. The findings highlight a significant shortfall in the practice of BSE among participants, despite its importance as an affordable, non-invasive, self-awareness tool to support early diagnosis of BC. The infrequent implementation of BSE practices aligns with other studies. Stefanut et al reported that 27% females practiced BSE at the baseline before implementing the intervention [41]. In addition, Zia Ullah et al. reported that 2.5% of females in the general population of Pakistan practiced BSE regularly, highlighting the need for improved education and training initiatives to establish BSE as a routine and accepted health practice [42]. Likewise, other studies conducted in the Pakistani context reported low practices infrequently among Pakistani females, that is, 18% reported by Raza et al [43] and 9.6% by Batool et al [44].

### Baseline Knowledge of BC and BSE

A thorough understanding of BC and BCS is essential for female compliance with BSE practice. The existing knowledge among Pakistani college teachers was assessed at the baseline of this study. The majority of teachers in the present study have limited information regarding breast cancer and breast self-examination. The findings of the present study align with the inadequate knowledge level among Pakistani women, as indicated by Ayesha Ahmed et al [45], which revealed a poor level of knowledge among female university students in Pakistan, that is 50%.

Moreover, the findings of a recent descriptive study conducted by Zonera et al, which assessed females from multiple cities in Pakistan, reported that 63.2% of females lacked knowledge of breast cancer [46]. Similarly, a study conducted among 135 women from a rural area of Lahore reported that only 19.3% of participants were aware of breast self-examination, while 28.9% had knowledge about breast cancer in general, and merely 24.4% knew how to perform breast self-examination [44].

Numerous studies have highlighted limited knowledge about breast cancer (BC) among women, frequently attributing this gap to the lack of structured educational programs. Researchers have constantly emphasized the necessity of breast cancer screening (BCS) activities to elevate awareness and enhance awareness using accessible media platforms [42,46,47].

The lack of organized breast cancer awareness and prevention programs is a major barrier to improving knowledge, particularly among college teachers in Pakistan who play key educational and social roles. Despite their influence and comprising nearly 60% of the teaching workforce [48], Female teachers at both junior and higher levels have been largely overlooked in research and interventions on breast cancer and BSE. While other populations have been studied, there is a notable gap in addressing awareness, attitudes, and preventive practices among this important group [43,49,50]. This oversight signifies a missed opportunity to engage in a demographic that may act as a catalyst for enhancing awareness and advocating for early detection methods in their educational institutions and wider communities.

Studies conducted at various locations, such as [51] in Saudi Arabia, in the UAE [52], in Ethiopia [53]Jordan also reported a breast cancer knowledge deficit among the teaching fraternity; similarly, research by Rabbani et al [54] reported that females residing in the UAE also showed lower knowledge scores for breast cancer and screening methods at their baseline assessments; these findings correspond with global patterns of insufficient knowledge.

Teachers are an essential yet often overlooked group in breast cancer awareness efforts. To harness their influence, policymakers, public health professionals, and educational institutions should work together to develop targeted strategies. Addressing key barriers such as limited time, fear, and lack of reliable information can empower teachers to become advocates for breast health, creating ripple effects that benefit not only themselves but also the wider communities they serve.

### Baseline beliefs associated with BC & BSE

This study identifies key gaps in health beliefs among Pakistani college teachers that hinder effective breast self-examination practices. The respondents revealed a low average score regarding their perceived susceptibility. This is inconsistent with the study conducted by [55], which reported lower perceived susceptibility scores at baseline. Low perceived susceptibility scores, in our study participants, indicate misconceptions about personal vulnerability to breast cancer, maybe arising from cultural beliefs or insufficient awareness of global risks [56]. The perceived barriers construct had a score of 12.0 (IQR 2.0) out of 20, indicating that participants acknowledged specific challenges but may lack approaches to address issues such as fear, shame, and time constraints.

Similar results are reported by the research conducted among females in Malaysia by Akhtari et al. [57] and research conducted in Nigeria [58]. The moderate severity score reflects a limited knowledge of the serious consequences of delayed diagnosis. Participants demonstrated higher confidence in executing BSE and moderate health motivation, indicating a favorable basis for intervention. However, if we relate the confidence to perform BSE with the Practice of BSE reported at baseline, the disparity is present; the disparity between elevated confidence in conducting breast self-examinations (BSE) and minimal actual practice among Pakistani college teachers. This highlights an intention-behavior gap, a common phenomenon in health behavior studies [59]. Although individuals assert statements such as “I can accomplish this,” their actions are hindered by multiple factors. Low perceived susceptibility indicates that individuals do not consider themselves at considerable risk. If individuals do not perceive themselves to be at substantial risk for BC, their confidence might not translate into consistent practice. Hence, diminishing their motivation to act in actuality.

Cultural and social obstacles, like stigma and discomfort over breast health, further discourage engagement, especially among confident individuals [60]. Practical barriers, like time constraints, competing goals, and fear around the revelation of any anomalies, increase this discrepancy. The lack of consistent reinforcement or reminders weakens the association between confidence and persistent behavior [61]. Interventions are essential to enhance self-efficacy, tackle risk perception, lessen stigma, and create supportive settings that encourage and sustain proactive health practices [62].

### Effectiveness of educational intervention

This randomized controlled trial showed that a well-designed educational intervention significantly improved teachers’ knowledge, beliefs, and practices related to breast self-examination. Post-intervention findings revealed marked progress in both understanding and behavior, highlighting the value of targeted education in addressing gaps. These results align with global evidence supporting the impact of focused health education, particularly in underrepresented communities [26, 63, 64].

### BSE Practice

The results indicate a significant improvement in BSE practice among the intervention group relative to the control group. The Generalized Estimating Equations (GEE) analysis indicated that at Time 2, the probability of engaging in BSE within the intervention group was significantly increased by a coefficient of 5.72 (95% CI: 2.644–12.39, p < 0.001) compared to the baseline. At Time 3, the probability increased markedly to 26.50 times (95% CI: 8.087–86.83, p < 0.001). Conversely, the control group showed no notable improvement with time, with odds ratios of 0.75 (95% CI: 0.470–1.220, p = 0.25) and 1.00 (95% CI: 0.584–1.714, p 1.0) for Time 2 and Time 3, respectively.

The significant improvement in the intervention group highlights the importance of transforming beliefs regarding health through focused educational campaigns. In a context like Pakistan, where breast cancer rates are rising [65]many women are aware of the disease but lack the guidance to take preventive action [5, 46]. This study’s participants, educated women, responded positively to the structured, hands-on intervention, which for most was their first exposure to practical BSE training using breast models. The interactive format encouraged questions and cleared up misconceptions, significantly boosting their understanding, confidence, and health beliefs. As a result, the improved BSE practices observed can be attributed to increased self-efficacy and awareness fostered by the intervention. These findings are inconsistent with the findings of the study, illustrating the link between HBM-based digital interventions and improved health practices. Initially, only 21% of participants performed BSE monthly. After using the app for three months, this figure increased to 68% (p < 0.001)[66].

This study’s findings align with an HBM-based intervention where monthly BSE practice rose from 24% to 70% (p < 0.001), highlighting that tackling cognitive, emotional, and behavioral barriers can effectively drive lasting preventive action [41].

This demonstrated that the transformation of perspectives through knowledge significantly improved BSE behaviors among women. The interactive and practical components of this study’s intervention correspond with concepts identified in prior research, emphasizing how hands-on guidance is more effective than passive methods like lectures or brochures [67].

While several gold-standard methods and techniques for cancer detection exist, they are more costly and necessitate the involvement of healthcare professionals and technicians, along with appropriate infrastructure. While breast self-examination (BSE) is no longer endorsed as a primary screening approach due to inadequate evidence regarding its impact on mortality, it remains an essential tool for promoting breast health awareness. Awareness of one’s breast helps in the early identification of abnormalities and encourages timely medical intervention in areas with limited resources, such as Pakistan [68]. This is in line with the recommendations released by the World Health Organization for BSE practice [69]. This highlights the significance of breast self-examination (BSE) in promoting awareness, as it is the simplest self-awareness tool rather than an absolute screening strategy.

### Knowledge/awareness of BC and BSE of research participants

The findings revealed the strong effectiveness of the intervention, as those who received it showed notable improvements in BC and BSE knowledge compared to pre-intervention scores. Conversely, the control group showed no notable changes within the same time frame. Furthermore, the GEE analysis for time, group, and the interaction term (time*group) revealed a significant interaction effect on knowledge scores (p < 0.001). The mean knowledge score in the intervention group increased by 15.78 points (CI: 15.10–16.47) at 1 month and by 16.64 points (CI: 16.02–17.27) at 3 months compared to the control group.

This improvement may be attributed to the structured and interactive format of the educational session, which provided clear and comprehensive information on breast cancer and BSE, addressing participants’ initial knowledge gaps. The significant improvements in knowledge highlight the role of targeted interventions in enhancing awareness and encouraging proactive health behaviors. In the Pakistani context, although many educated women acknowledge the rising burden of breast cancer, their understanding of the disease and BSE often remains limited or inaccurate. The effectiveness of this intervention demonstrates the importance of delivering precise, evidence-based content through structured programs that include discussions, practical training, gamified learning, and myth correction, enabling participants to dispel misconceptions and develop practical skills in a supportive environment.

These results align with a quasi-experimental study where HBM-based education raised adequate knowledge of BC and BSE from 39% to 83% (p < 0.001), confirming the impact of structured health education on women’s preventive awareness [41]Similarly, a 2021 quasi-experimental study found that HBM-based smartphone education raised women’s knowledge of BC risk factors and BSE from 36% to 81% (p < 0.001), showing the effectiveness of accessible, repeatable learning tools [70]

Likewise, research conducted in Bangladesh [24], in India and Iran [67, 71, 72], indicated that educational interventions significantly strengthen knowledge scores in the intervention groups relative to the control groups (p < 0.001).

### Beliefs of BC and BSE

#### Perceived Susceptibility.

Perceived susceptibility significantly increased at both follow-ups (p < 0.001), with GEE showing strong time*group interaction effects. Notably, scores increased at one month (β = 2.947) and further at three months (β = 3.298), reflecting the intervention’s lasting impact on breast cancer risk awareness in a culturally influenced population.

A key challenge in Pakistan is the deeply ingrained misconception that breast cancer primarily affects older women or those with a hereditary predisposition, leading to a diminished sense of personal risk among younger women. This false perception often results in delayed screening behaviors and reluctance to adopt preventive measures such as breast self-examination (BSE) [2]. The intervention directly targeted these barriers by delivering structured, evidence-based education through a combination of PowerPoint presentations, testimonial videos, interactive discussions, human breast models, and question-and-answer sessions, tailored to address the specific sociocultural context of Pakistani females.

These findings support a study using an HBM-based mobile app, where perceived susceptibility increased from 29% to 67% (p < 0.001), highlighting the effectiveness of tailored risk information in raising personal risk awareness [70]. The improvement in perceived susceptibility reflects a lasting cognitive shift initiated by the intervention. Similar findings were reported in Ethiopia, where HBM-based education raised perceived susceptibility from 28.5% to 65.2% (p < 0.001) among female students [73]. This aligns with findings from earlier studies [24, 30, 74], which highlight that targeted educational initiatives significantly enhance risk perception and preventive health behaviors (p < 0.001), particularly in populations with low baseline awareness.

#### Perceived Severity.

The intervention group exhibited significant improvements in perceived severity scores at both the 1-month and 3-month follow-ups (p < 0.001). GEE analysis shows that perceived severity scores significantly increased in the intervention group over time, with a rise of 3.58 points at one month (β = 3.579, CI: 3.090–4.068, p < 0.001) and 5.44 points at three months (β = 5.439, CI: 4.839–6.038, p < 0.001). No significant difference was found at baseline (p = 0.786), and the control group showed no notable change.

One of the most influential elements of the intervention was the inclusion of one of the breast cancer survivor teacher narratives, which shared compelling real-life experiences illustrating the consequences of delayed diagnosis and treatment. These testimonies personalized the risk, making breast cancer more tangible and relatable, consistent with evidence that real-life stories evoke stronger emotional responses than statistics alone [75].

To reinforce perceived severity, the module highlighted risk factors, stages, complications, and survival without explicit fear appeals, but by discussing late-stage consequences and Pakistan’s high mortality rates, it underscored the urgency of early detection. This contextual framing, combined with demonstrations of disease progression and survivor stories, helped participants grasp the seriousness of the disease while equipping them with practical knowledge to act proactively.

However, the intervention ensured that fear was used constructively, avoiding defensiveness by pairing fear-based content with actionable strategies such as BSE training. This aligns with the Extended Parallel Process Model (EPPM which suggests that fear appeals are most effective when accompanied by self-efficacy-enhancing strategies [76].

This study supports HBM-based findings from the previous study, where perceived severity increased from 40% to 84% (p < 0.001) after app-based videos and survivor stories, showing that real-world narratives effectively strengthen awareness of breast cancer’s seriousness [70].

#### Perceived benefits.

Perceived benefits significantly increased in the intervention group at one month (β = 2.281) and three months (β = 3.579) post-intervention (p < 0.001), with no change in the control group. This underscores the intervention’s role in promoting early detection and encouraging proactive health behavior.

These results concur with the observations of the study, in which HBM-guided education was provided to female students in Northwest Ethiopia to improve preventive breast health behaviors. Before the sessions, only 37.5% believed BSE was truly beneficial for early detection; this figure climbed to 85.7% afterward (p < 0.001), showing that the program effectively demonstrated BSE’s value [73].

These results are consistent with the findings from a randomized trial that highlighted the benefits of doing BSE regularly. Many Yemeni teachers were unsure about its value before the session; only 40% believed BSE could help find breast cancer early. The HBM sessions used examples and group discussions to explain benefits. After the intervention**,** 86% agreed on its usefulness (p < 0.001).

#### Perceived Barriers.

Perceived barriers to BSE significantly declined in the intervention group at one month (β = 3.351) and three months (β = 3.491) post-intervention (p < 0.001), with no change in the control group, highlighting the intervention’s success in reducing obstacles to practice.

These findings align with an RCT in Yemen, where HBM-based education reduced reported BSE barriers from 56% to 25% (p < 0.001), showing that addressing emotional and cultural concerns boosts women’s confidence in practicing BSE [30].The findings align with the results of Olgun [77], which similarly reported a significant reduction in perceived barriers following educational interventions (p < 0.005).

#### Self-Efficacy.

Self-efficacy significantly improved in the intervention group at one month (β = 4.053) and further at three months (β = 5.070) post-intervention (p < 0.001), with no change in the control group, highlighting the intervention’s strong impact on confidence to perform BSE.

This increase highlights the value of hands-on, structured training in building women’s confidence to perform breast self-examination (BSE). Since low self-efficacy is a known barrier to regular practice, this improvement signals a meaningful impact. A key strength of the intervention was its experiential approach, combining step-by-step guidance with gamified learning and breast model practice. This not only improved technique but also offered real-time feedback and clarity, fostering the confidence needed for long-term commitment to BSE.

These findings align with Noman et al. [30], Sun et al. (2019) [78]. This substantial gain illustrates how practice and training, when grounded in the HBM, can empower women with both the knowledge and confidence needed for effective self-care [74]. These findings align with a study where BSE barriers dropped from 52% to 23% (p < 0.001) post-intervention, demonstrating that HBM-based modules effectively reduce emotional and practical obstacles to self-care [41]. Similarly, Ashtariran et al [37], emphasize that practical training significantly enhances self-efficacy and confidence in performing BSE (p < 0.001).

#### Cues to action.

Similarly, for health motivation scores, the average improvement was significant, rising after one and three months (p < 0.001) post-intervention. Moreover, GEE results show a significant interaction effect for cues to action, with the intervention group showing an increase at one month (β = 1.561, CI: 0.969–2.154, p < 0.001) and a larger rise at three months (β = 4.912, CI: 4.097–5.728, p < 0.001). No significant baseline difference (p = 0.710) or change in the control group was observed.

These findings match those of Ahmadifaraz and Jouzi (2024), who found that using reminders helps motivate women to do BSE regularly. In the Najafabad study, only 36% of students had any reminders for BSE at first. After joining group sessions with reminders, role-plays, and peer help, this went up to 79% (p < 0.001). This shows that group-based reminders and activities can help women keep up with regular self-exams [74].

This increase indicates a heightened awareness of the importance of preventative measures, health behaviors, and self-examination. Motivation to perform BSE may have been enhanced by external cues, such as WhatsApp reminders and messages highlighting BSE steps and breast cancer symptoms. These served as prompts that reinforced awareness and encouraged regular practice, aligning with the cues to action component of the Health Belief Model. Similarly, survivor testimonials included in the intervention module and interactive talks highlighting the consequences of delayed diagnosis were essential in developing motivation. These results correspond with a study conducted by [79], which documented similar improvements in health motivation after educational programs.

## Conclusion

This study shows that a Health Belief Model–based intervention significantly improved breast cancer awareness, beliefs, and BSE practices among female college teachers in Hyderabad. By combining structured education with hands-on training, the program enhanced knowledge, increased self-efficacy, reduced barriers, and led to sustained behavioral change. These findings support the value of theory-driven, practical education in promoting early detection and empowering women’s health in underserved communities.

### Strength

This study has several key strengths. The use of a cluster-randomized controlled trial design minimized contamination between groups and strengthened internal validity. Applying the Health Belief Model and using a validated tool to assess knowledge, beliefs, and practices ensured both theoretical and measurement rigor. Stratified sampling of faculty enhanced representativeness, while a high response rate with no dropouts preserved group balance and study power. Additionally, the use of Generalized Estimating Equations allowed for accurate analysis by accounting for clustering and confounding, reinforcing the reliability of the findings.

### Limitation

Despite its strengths, this study has some limitations. It focused solely on female college teachers in Pakistan, which may limit generalizability to women from other backgrounds. Additionally, a modest sample size may affect the precision of some findings. Future research should include larger, more diverse samples to enhance relevance and validity. Nonetheless, this study offers important evidence on the effectiveness of educational interventions in improving breast cancer awareness and BSE practices, providing a foundation for future efforts to strengthen screening adherence in similar contexts.

## Supporting information

S1 Checklist CONSORT-2010-Checklist (1). (DOC)

S1 Protocol PROTOCOL. (DOCX)
